# Immunoradiotherapy as an Effective Therapeutic Strategy in Lung Cancer: From Palliative Care to Curative Intent

**DOI:** 10.3390/cancers12082178

**Published:** 2020-08-05

**Authors:** Rodolfo Chicas-Sett, Juan Zafra-Martin, Ignacio Morales-Orue, Juan Castilla-Martinez, Miguel A. Berenguer-Frances, Elisa Gonzalez-Rodriguez, Delvys Rodriguez-Abreu, Felipe Couñago

**Affiliations:** 1 Department of Radiation Oncology, Dr Negrin University Hospital of Gran Canaria, Barranco de la Ballena S/N, Planta 2, 35010 Las Palmas de Gran Canaria, Spain; jzafmar@gobiernodecanarias.org; 2 Department of Radiation Oncology, IMED Hospitales, Avenida Nueva Condomina 11, 30110 Murcia, Spain; iemorales@imedhospitales.com (I.M.-O.); jfcastilla@imedhospitales.com (J.C.-M.); 3 Department of Radiation Oncology, Nisa-Vithas Virgen del Consuelo Hospital, Callosa d’en Sarrià 12, 46007 Valencia, Spain; maberenguer@iconcologia.net; 4 Department of Medical Oncology, Complejo Hospitalario Universitario Insular Materno-Infantil de Gran Canaria, Calle Francisco Hernández González 1, 35016 Las Palmas de Gran Canaria, Spain; elisaglezrguez@gmail.com (E.G.-R.); delvys.rodriguez102@alu.ulpgc.es (D.R.-A.); 5 Clinical Deparment, Faculty of Biomedicine, Universidad de las Palmas de Gran Canaria, Calle Juan de Quesada 30, 35001 Las Palmas de Gran Canaria, Spain; 6 Department of Radiation Oncology, Quirónsalud Madrid University Hospital, Calle Diego de Velázquez 1, 28223 Pozuelo de Alarcón, Madrid, Spain; felipe.counago@quironsalud.es; 7 Department of Radiation Oncology, La Luz Hospital, Calle Maestro Ángel Llorca 8, 28003 Madrid, Spain; 8 Clinical Deparment, Faculty of Biomedicine, Universidad Europea de Madrid, Calle Tajo, S/N, 28670 Villaviciosa de Odón, Madrid, Spain

**Keywords:** lung cancer, radiotherapy, immunotherapy, immune-checkpoint inhibitors, abscopal effect

## Abstract

Lung cancer is one of the main causes of cancer-related mortality worldwide. Over the years, different therapeutic modalities have been adopted depending on tumor stage and patient characteristics, such as surgery, radiotherapy (RT), and chemotherapy. Recently, with the development of immune-checkpoint inhibitors (ICI), the treatment of metastatic and locally advanced non-small cell lung cancer (NSCLC) has experienced a revolution that has resulted in a significant improvement in overall survival with an enhanced toxicity profile. Despite this paradigm shift, most patients present some kind of resistance to ICI. In this setting, current research is shifting towards the integration of multiple therapies, with RT and ICI being one of the most promising based on the potential immunostimulatory synergy of this combination. This review gives an overview of the evolution and current state of the combination of RT and ICI and provides evidence-based data that can improve patient selection. The combination in lung cancer is a safe therapeutic approach that improves local control and progression-free survival, and it has the potential to unleash abscopal responses. Additionally, this treatment strategy seems to be able to re-sensitize select patients that have reached a state of resistance to ICI, further enabling the continuation of systemic therapy.

## 1. Introduction

Lung cancer is one of the main causes of cancer-related mortality worldwide. The most frequent histological subtype, with up to 84% of cases, is non-small cell lung cancer (NSCLC) [1]. With the historically available multimodal treatments, the 5-year survival rate for metastatic patients has been no higher than 5% [2,3,4].

The technological advances of radiotherapy (RT) have allowed for the administration of high doses of radiation with great precision and low rates of toxicity. This was first evidenced with the use of Stereotactic Ablative Radiotherapy (SABR) in early stage inoperable patients, which achieved comparable results to surgery in terms of local control (LC). Even with these positive results, about 15–20% of these patients present distant failure after two years [5]. This data reinforces the idea that advances in this setting must not come from the intensification of current therapies but through the integration of new treatments based on the biology of the tumor.

The introduction of immune checkpoint inhibitors (ICI) has been a paradigm shift in the standard of care (SoC) for lung cancer, mainly in NSCLC [6,7,8]. Despite the good outcomes achieved with ICI, most lung cancer patients experience primary resistance to immunotherapy, which is currently the most critical challenge in this setting. Moreover, the development of secondary resistances in initially responding patients is also a relevant problem. In this regard, there is growing evidence that RT is a key contributor to antitumor response, which supports the idea that the immunostimulatory effects of RT can be exploited in order to augment the systemic response to ICI [9].

This review considers the evolution of the use of RT in combination with ICI in lung cancer from its beginning and up to contemporary practice. The aim was also to provide information that can improve patient selection in order to maximize the benefit of this treatment approach.

## 2. The Rationale for the Use of RT

RT has a key role in the treatment of lung cancer in all its stages [10]. In Europe, over 60% of NSCLC patients have advanced disease at diagnosis, a majority of whom are treated with chemotherapy (CT) and palliative support exclusively [11]. In this regard, the use of palliative RT has been traditionally based on providing symptomatic relief in order to improve quality of life [12]. However, about 20–50% of stage IV patients present a limited number or metastases (what is commonly known as oligometastatic state) [13,14]. In this setting, the management of these patients includes local ablative therapies, such as SABR. Several studies, mostly retrospective, have suggested that administering aggressive local treatments to all metastatic lesions improve both progression-free survival (PFS) and overall survival (OS). This retrospective data has been reinforced through two randomized phase II trials. Gomez et al. [15] randomized stage IV NSCLC patients with no progression after 3 months of first-line therapy to receive local consolidative therapy (RT or surgery) to 3 or fewer metastases versus observation/maintenance treatment. OS was 41.2 months in the experimental arm versus 17 in the control. Similar outcomes were reported by Palma et al. in the SABR-COMET trial [16]. Ninety-nine patients were analyzed, 33% of which were assigned to the control group (who received SoC treatment) and 67% to the SABR group (SABR to all known metastatic sites). With a median follow-up of 51 months, median OS was 28 months in the control group vs. 50 months in the SABR group. As a follow-up to that study, a phase III trial (SABR-COMET-10) evaluating the impact of SABR in subjects with 4–10 metastatic sites is currently ongoing. The results of the SABR-COMET trial emphasize the value of SABR in selected stage IV lung cancer patients, and its follow-up study might shed some light on the true potential of SABR in the metastatic setting.

In locally advanced disease, RT associated to CT is the SoC for patients with inoperable or unresectable stage III disease [3]. A meta-analysis of several studies evaluating the combination of RT and platinum-based CT showed improved OS when these were administrated concurrently compared with a sequential regime [17].

Lastly, in early stages, SABR is the SoC for medically inoperable patients. Overall, SABR is an effective treatment in this setting, with LC rates of 90–95% (comparable to lobectomy) [2].

## 3. Immunotherapy in Lung Cancer

Recent progress in the understanding of the crosstalk that occurs between immune cells and tumor cells has led to the identification of potential targets to control tumor growth. Drugs targeting immune checkpoints of this interaction are monoclonal antibodies known as ICI and have become a cornerstone in NSCLC. ICI target two major receptors. Firstly, cytotoxic T lymphocyte-associated antigen-4 (CTLA-4), which is expressed on the surface of T lymphocytes and transmits an inhibitory signal that downregulates T-cell activation. CTLA-4 blockade removes this signal and T-cell activation is consequently enhanced. To date, none of the CTLA-4 blockers in monotherapy have demonstrated significant results in NSCLC. The second-generation ICI mechanism is directed towards programmed cell death protein 1 (PD-1) or its ligand PD-L1. PD-1 is a negative regulatory receptor expressed by T and B lymphocytes and natural killer cells. Its role consists of limiting the response of these cells in order to protect healthy tissues. Therefore, by blocking PD-1 we can “lift the brakes” of these cells and enhance the immune system. The anti-PD-1 drugs approved for NSCLC are pembrolizumab and nivolumab, whereas atezolizumab and durvalumab target PD-L1. The number of authorized indications has been growing ever since the first approval, both in the advanced disease and adjuvant settings and across all histologies (Table 1).

Currently, ICI is part of the SoC in metastatic NSCLC. In 2015, nivolumab, which was already approved for melanoma, demonstrated improved OS in metastatic NSCLC both in squamous and non-squamous as second-line treatment (CheckMate017, CheckMate057) [18,19]. Subsequently, both pembrolizumab and atezolizumab reported prolonged OS as second-line therapy (Keynote-010, OAK trial) [20,21]. The Keynote-010 was the first trial that selected patients with a PD-L1 expression in tumor cells ≥ 1%. Those patients with a higher expression of PD-L1 tended to have better responses. ICI were then tested as first-line treatments. Pembrolizumab was compared to platinum-based CT in NSCLC with PD-L1 ≥ 50%, a highly selected population based on the results in second-line. Keynote-024 in 2016 significantly improved OS (10.3 vs. 6.0 months, HR 0.60), which led to the approval as first-line treatment [7]. Only two years later, the Keynote-189 and Keynote-407 trials (in non-squamous and squamous NSCLC, respectively) assessed the efficacy of the combination of platinum-based CT and pembrolizumab, which significantly prolonged OS (KN-189 not reached vs. 11.3 months, HR 0.49; KEYNOTE-407 15.9 months vs. 11.3 months, HR 0.64) [22,23]. This effect was independent of the expression of PD-L1, including those with no expression at all. Combination of platinum-based chemotherapy and pembrolizumab is currently the standard first-line treatment for fit patients.

In the adjuvant setting, the PACIFIC study evaluated the effect of maintenance therapy with durvalumab following chemoradiotherapy (CRT) for unresectable stage III NSCLC [8]. PFS was drastically improved (16.8 vs. 5.6 months, HR 0.52) and became a new SoC.

NSCLC carrying mutation drivers have not been particularly responsive to ICI, possibly due to their characteristically lower mutational burden. An exception is the combination of carboplatin, pemetrexed, bevacizumab and atezolizumab [24]. In the ImPower150 trial, PFS among patients with *EGFR* mutations or *ALK* translocations was longer with the combination with atezolizumab compared to the combination without atezolizumab, achieving a PFS increase of 3.6 months (median, 9.7 months vs. 6.1). This allows for a more effective treatment once targeted therapies have failed.

In small cell lung cancer (SCLC), ICI monotherapy has achieved modest results. However, in the ImPower133 study, the combination of atezolizumab with the standard carboplatin and etoposide has recently shown significant benefit [25]. After a median follow-up of 13.9 months, median OS was higher for the atezolizumab arm (12.3 months [95% CI 10.8–15.9] vs. 10.3 months [95% CI 9.3–11.3]). Median PFS significance was also met with 5.2 months in the atezolizumab arm compared with 4.3 months in the placebo group (HR 0.77, 95% CI 0.62–0.96, *p* = 0.017). The addition of atezolizumab doubled 12-month PFS rate (12.6% vs. 5.4%). This combination scheme is the first one to increase survival in extended disease SCLC in decades and is now a new long awaited SoC.

The mechanism of action of ICI determines a range of toxicities that differs from those seen with classic cytotoxics. By unleashing brakes and promoting the activity of the immune system, immunologic tolerance can be altered, triggering reactions mediated by self-directed antigens, known as immune-related adverse events (irAEs). The most frequently affected tissues are the skin, the gastrointestinal system, and the endocrine glands [26]. However, irAEs have been described in almost any tissue, including those with immune privilege, such as the central nervous system, the myocardium, or the kidneys [27]. Accordingly, physicians must keep a high grade of suspicion and seriously evaluate every new symptom that arises. Although most irAEs are mild, steroids are the cornerstone when treatment is needed (grade ≥ 2). Multidisciplinary management of severe toxicities is mandatory.

## 4. The Role of Radiation in the Immune Response to Cancer

The biological mechanisms that explain the local antitumoral effect of RT have been well established for decades [28]. In short, radiotherapy induces DNA damage that results in the interruption of the cell cycle, leading to the death of tumor cells via apoptosis and necrosis [29].

However, in recent years, research is shifting towards the added effects that RT has outside the radiation field, which seem to be immune related and might explain what is known as the abscopal effect (AE) [30]. This concept was initially coined by Mole in 1953 when describing a systemic antitumoral action after local RT that can result in distant responses [31]. This new research is allowing for a better and wider understanding of these immune mechanisms that can have consequences beyond the site of radiation.

**Figure 1:**
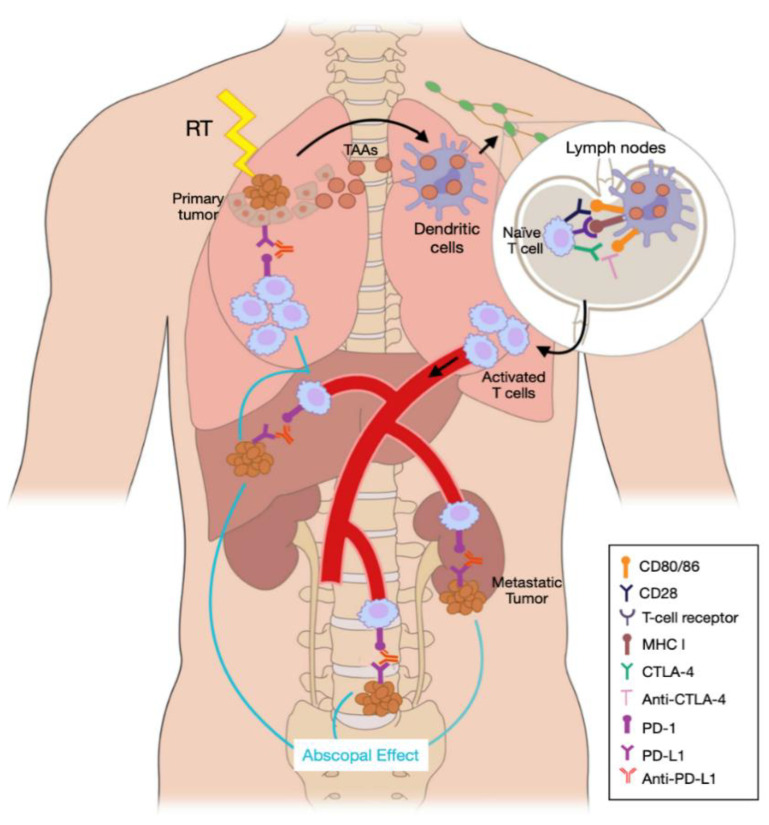
When delivered to the tumor, radiotherapy (RT) induces immunogenic cell death and the release of tumor-associated antigens (TAAs) nearby. Dendritic cells (DC) recognize these TAAs and carry them to the lymph nodes, where they present them to naïve CD8^+^ T cells through the major histocompatibility complex I (MHC I) and CD80/86 and CD28 receptors. At this point, anti-cytotoxic T lymphocyte-associated antigen (CTLA)-4 agents block the CTLA-4 receptor in naïve T cells, which ceases their inhibition. Activated cytotoxic T cells are then incorporated into the bloodstream and travel to distant metastases and back to the irradiated tumor to eliminate the disease. At this stage, anti-PD-1/L1 therapy blocks the interaction between these two receptors, which allows for a stronger antitumor effect driven by T cells.

Although much is still unknown, it has been established that RT induces immunogenic cell death (ICD) by releasing multiple tumor-associated antigens (TAAs), chemokines, inflammation mediators and other immune related molecules. Each of these can have either immunostimulatory or immunosuppressive effects, with a general trend towards increased stimulation of the immune system [32,33]. The main immunostimulatory substances activated after RT include damage-associated molecular patterns (DAMPs), high mobility group box 1 (HMGB1), heat shock proteins, interferon type I (IFN-I), and interferon gamma (IFN-γ), among others. These promote the maturation of dendritic cells (DC) and the presentation of TAAs to these DC, which then migrate to the lymph nodes and present these antigens to naïve CD8+ T cells through the major histocompatibility complex I (MHC I). As a result, RT serves as an in-situ vaccination that primes and activates antigen-specific cytotoxic T cells that are then ready to enter the circulation and search for the tumor both inside (as RT also facilitates access to the tumor by remodeling its vascularity) and outside the site of irradiation [34,35,36]. A graphical representation of this phenomenon can be seen in Figure 1. However, it must be noted that RT also induces the activation of immunosuppressive molecules, such as transforming growth factor beta (TGF-β), which is a critical factor for regulatory T cell (Treg) differentiation [37]. These suppressive substances are partially regulated by stimulator of interferon genes protein (STING) and C-C chemokine receptor type 2 (CCR2). The pathways in which these substances are involved are being investigated for their role in the resistance to RT [38]. The balance of these immunostimulatory and immunosuppressive substances varies depending on dose, fractionation, and other treatment and tumor variables, and it might be the key to determining the overall influence of radiation in the immune system [39].

Although the immune mechanisms unleashed by RT are becoming more understandable, the development of an AE is still a rare event. However, since the introduction of ICI, multiple preclinical studies have found that its combination with RT increases the chances of obtaining an AE [40,41]. It is known that tumors can escape the immune response by the upregulation of PD-1 ligands, which cause T cell exhaustion. Anti-PD-1/L1 antibodies inhibit this blockade, favoring a stronger T cell antitumoral effect [42]. On the other hand, anti-CTLA-4 antibodies both inhibit Treg and stop CTLA-4 from binding to CD28 and inhibiting T cell activation, also resulting in an increased immune response [43].

## 5. Immunoradiotherapy in Metastatic Lung Cancer

One of the reasons why lung cancer is among the tumor types with highest mortality rates is that most patients are metastatic at the moment of diagnosis. In fact, the prognosis of patients with metastatic NSCLC is very poor, with a median OS of less than seven months [44]. As mentioned before, the irruption of ICI has revolutionized the treatment landscape of NSCLC, which has had a significant impact on OS. However, primary and secondary resistances to ICI remain a relevant problem in most patients [20,45,46].

The preclinical evidence on the immunomodulatory effect of RT, as well as the positive results of SABR in terms of LC, OS, and PFS in oligometastatic patients [16], has pushed clinicians to reconsider the role of RT in the metastatic stage, evolving from a purely palliative care to its integration with ICI in order to improve systemic responses and unleash the AE [9,47]. This rare phenomenon, however, does not escape controversies. Up to 2019, only six cases had been described in NSCLC, and four of them had received treatment with ICI. Moreover, some authors have questioned the existence of the AE in patients with ICI, given that this effect was first described when RT was delivered in absence of an additional systemic treatment. Most studies in this field tend to consider the improvement in overall response rate (ORR) observed after the association of RT to ICI as a surrogate for AE, which adds to the confusion. For this reason, other authors have preferred to adopt the term of ‘systemic therapy augmented by radiotherapy’ (STAR) when assessing the enhancement of the systemic effects of ICI after the addition of RT [48].

### 5.1. How did the Preclinical Evidence for the Combination of Ici and Rt Translate into the Clinical Setting?

The combination of ICI and RT in NSCLC started to gain more interest after the publication of the KEYNOTE-001 study. A secondary analysis of the 97 patients included in this phase I trial showed that 43% had been treated with RT prior to the administration of pembrolizumab. PFS was longer in those patients who had previously received RT (4.4 vs. 2.1 months). Moreover, a benefit in OS was also observed in this subgroup (11.6 vs. 5.3 months) [49].

These interesting results led to the analysis of further retrospective data in order to find if these benefits could be replicated. In this regard, both Desideri and Ratnayake found similar results in NSCLC patients, with a trend towards a doubling in PFS and OS in those who had received RT and ICI [50,51]. Despite this encouraging data, several new questions arose with respect to timing, dose escalation and other treatment variables. In terms of timing, some light was shed by Samstein et al. In this retrospective study, 758 patients who received ICI and RT were analyzed. A benefit in OS was observed in those patients who received these treatments concurrently vs. those who received them separately. Furthermore, patients who had RT administered at least one month after the first dose of ICI had a significantly higher OS compared to those who received RT less than one month after the start of ICI (20 vs. 11 months) [52].

### 5.2. Can This Retrospective Data Be Replicated in Clinical Trials?

The first prospective studies that were designed to evaluate the AE have the disadvantage of including a very heterogenous group of patients with different tumor histology, RT dose and treatment sequence. For instance, the study by Tang et al. assessed 35 patients (8 with NSCLC) and found that, by associating SABR to a metastatic lung or liver lesion with anti-CTLA-4, 23% of patients experienced a response in the non-irradiated lesions [53].

Formenti et al. designed a trial in a more controlled setting. This phase I-II study included 39 patients with metastatic NSCLC who received four cycles of ipilimumab in combination with a SABR regime of 28.5 Gy in 3 fx or 30 Gy in 5 fx (based on the fractionation that showed synergy with anti-CTLA-4 in preclinical studies). A 31% disease control was reported which, at that moment, showed a promising clinical benefit with this combined approach in metastatic NSCLC [54]. However, it was necessary to confirm this benefit in terms of ORR, PFS and OS. To this end, Welsh et al. conducted a phase II study combining anti-CTLA-4 with SABR doses of 50–60 Gy in 4–10 fx. Although this trial was limited by the fact that it included different tumor types, it must be noted that those patients with NSCLC obtained a more significant clinical benefit, with an ORR of 67%. Overall, PFS was 5 months and OS 12 months, considering all tumor histologies [55].

Given the benefit of the combination, a prospective study is trying to determine the rate of AE separated from the ORR by preselecting lesions outside of the radiation field and evaluating their behavior after SABR to the target lesions. Preliminary results are promising, with an AE rate of 33% and an ORR of 53%. Of particular interest is the fact that, in this study, all patients had failed to ICI monotherapy but could maintain this same treatment until new progression by adding SABR [56].

These first results of the combination of SABR and ICI, now commonly known as I-SABR, are finally starting to gain enough interest in order to develop randomized studies that compare this approach with a control arm of exclusive ICI (Table 2). The recent PEMBRO-RT is a phase II randomized trial that included 76 patients with metastatic NSCLC in which the experimental arm received pembrolizumab plus SABR (24 Gy in 3 fx) to a single lesion. After a follow-up of 24 months, ORR at 12 weeks was 36% in the experimental arm versus 18% in the ICI monotherapy group. Disease control was also higher in the I-SABR group (64% vs. 40%, *p* = 0.04). Median PFS was 6.6 months versus 1.9 months. A tendency towards an improved OS, though not statistically significant, was also observed (15.6 months in the I-SABR arm vs. 7.6 months in the control arm). Interestingly, patients with PD-L1 < 1% presented a higher clinical benefit with the addition of SABR. The results of this study support those obtained in retrospective and prospective series, although its primary endpoint of an ORR of 50% in the experimental arm was not achieved [57].

Another randomized phase I/II study of 72 patients (36 per arm) is of particular interest due to the fact that it compared two regimes of RT in the experimental arm: either SABR (50 Gy in 4 fx or 70 Gy in 10 fx) or conventional RT (45 Gy in 15 fx) associated to pembrolizumab. In a sub-analysis of the experimental arm, AE in the SABR group was 38% versus 10% in the conventional RT group (although not statistically significant). Moreover, the SABR group reported a PFS of 21.1 months versus 6.8 months in the conventional RT arm (*p* = 0.03). This study reinforces the importance of RT dose in order to stimulate the immune response [58].

Finally, a phase I study of 35 patients receiving SABR randomized them in two arms: concurrent or sequential dual ICI (ipilimumab plus nivolumab). PFS was 6.2 months in the sequential arm and 5.9 in the concurrent arm. Total ORR was 68% [59].

**Table 2:** Selected randomized and prospective trials testing the combination of RT and ICI in metastatic NSCLC patients.

Author/Trial	Phase	N	Treatment Arms	ORR (%)	Median PFS (months)	Median OS (months)	irAEs ≥ G 3 (%)
Theelen et al. [57]/PEMBRO-RT	II randomized	76	SABR + pembrolizumab Pembrolizumab	36 vs. 18	6.6 vs. 1.9	15.6 vs. 7.6	11
Welsh et al. [55]	II randomized	72	SABR/Conventional RT + pembrolizumab Pembrolizumab	22 vs. 25 *	10.9 vs. 8.4 *	NR	15
Patel JD et al. [59] COSINR	I randomized	35	SABR + concurrent ipilimumab/nivolumab SABR + sequential ipilimumab/nivolumab	68 (total)	6.2 vs. 5.9	NR	11
Bauml et al. [60]	II	45	Locally ablative therapy (surgery/SABR) + pembrolizumab	NR	19.1	41.6	10
Formenti et al. [54]	I/II	39	SABR + ipilimumab	31	7.1	13.0	10.3

* In the sub-analysis of the RT arm (SABR vs. conventional RT): overall response rate (ORR) 38% vs. 10%, progression-free survival (PFS) 21.1 months vs. 6.8 months. SABR: stereotactic ablative body radiotherapy. RT: radiotherapy. NR: not reported.

For this review, only studies in which lung cancer was the major or the exclusive histology have been assessed. However, global results are comparable with those reported in a systematic review that included both retrospective and prospective series of NSCLC patients, but also other tumor types. The mentioned review reported a global rate of AE/ORR of 41% [9]. A great number of trials combining ICI and RT are ongoing. The most relevant are described in Table 3. Of particular interest is a randomized phase III trial (NCT03867175) that will compare ICI monotherapy with I-SABR to all metastatic lesions and may be able to confirm the OS benefit suggested in previous studies.

### 5.3. Could the Safety Profile of the Combination Be an Issue?

While evidence on I-SABR is still limited, the available data suggest that toxicity derived from this combined treatment does not increase in comparison to immunotherapy alone in the metastatic setting. A recent systematic review showed grade ≥ 3 median toxicity rates of 14.5% with anti-PD-1/L1 plus SABR and 26% with anti-CTLA-4 plus SABR [9]. In addition, the PEMBRO-RT trial only reported a 11% rate of pneumonitis in the I-SABR arm [57]. Furthermore, the phase II study by Bauml showed comparable rates of toxicity [60]. Even with dual ICI plus SABR, the study by Patel et al. only showed 11% of dose limiting toxicity [59].

## 6. Immunoradiotherapy in Locally Advanced NSCLC

Stage III NSCLC represents a heterogeneous group of patients with variable prognosis. For early resectable stage III NSCLC, which accounts for approximately 20–30% of patients [61], surgery is the primary curative treatment, which is usually accompanied by neoadjuvant and/or adjuvant CT and RT, resulting in 5-year OS rates of 50–70% [62]. However, in more advanced cases, surgery is rarely feasible and, in turn, definitive concurrent CRT is the SoC [63], with 5-year OS rates of approximately 15–20% [64].

**Figure 2:**
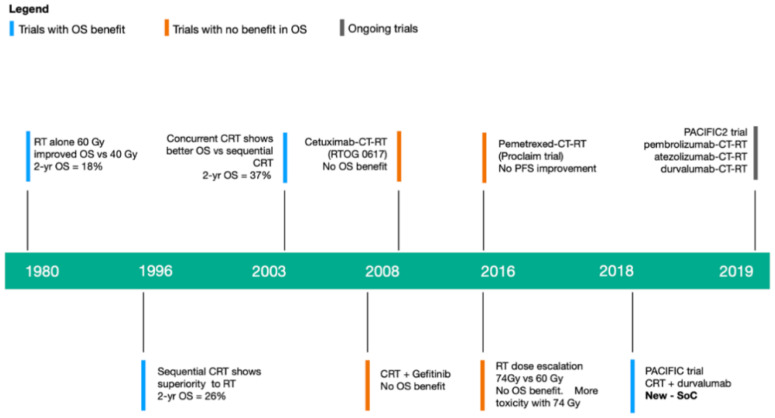
Timeline illustrating the evolution of treatments for NSCLC. RT: radiotherapy. CT: chemotherapy. CRT: Chemoradiotherapy. OS: overall survival. PFS: progression-free survival. Yr: year. SoC: standard of care.

Given that these results are not optimal, several approaches have tried to improve the outcomes of concurrent CRT. Induction or consolidation CT have failed to improve PFS and OS [65,66,67,68]. Furthermore, greater radiation doses up to 74 Gy compared with the standard 60 Gy have led to more side effects and reduced survival [3,69]. Additional randomized phase IIb and phase III trials of consolidation therapy with vaccines and targeted therapies (cetuximab, gefitinib, etc.) have shown no differences regarding OS or PFS [70,71,72,73]. Therefore, recent efforts to improve outcomes in stage III NSCLC have shifted towards new strategies that integrate ICI in the current regimes, first as consolidation therapy after CRT, and more recently in the definitive and neoadjuvant settings. This evolution in the treatment of stage III NSCLC is represented in Figure 2.

### 6.1. What Is the Evidence for Administering ICI Consolidation Therapy?

Interest in the addition of ICI to conventional therapies has greatly increased since the publication of a landmark study in 2018: the PACIFIC trial (Table 4). This was the first double-blind, randomized phase III trial that evaluated the use of the ICI durvalumab for 12 months after definitive CRT in patients with unresectable NSCLC and no disease progression after CRT. For the 714 patients included, durvalumab achieved a median PFS of 16.8 months compared to 5.6 months with placebo [8]. The durvalumab group also had a higher response rate (28.4% versus 16%). In terms of OS, the durvalumab group showed a 24-month OS of 66.3% vs. 55.6%, whereas median OS had not been reached. It must be noted that these improved results were independent of PD-L1 expression. Durvalumab was also well tolerated. 30.5% of the patients in the durvalumab group and 26.1% in the placebo group had grade 3 or 4 adverse events of any cause. 15.4% vs. 9.8% of patients discontinued the trial regimen because of adverse events [74].

Subsequent post-hoc and subgroup analyses of the PACIFIC trial have detailed some of these results, showing improved PFS and OS in the durvalumab group regardless of CT type, radiation dose or time from radiation to randomization [75]. Moreover, the updated 3-year OS rates presented at the 2019 meeting of the American Society of Clinical Oncology (ASCO) showed a 36-month OS rate of 57% in the durvalumab group vs. 43.5% in the placebo. Median OS had still not been reached [76]. These encouraging results have led to the establishment of consolidation therapy with durvalumab as a new SoC in unresectable stage III NSCLC.

Interestingly, one of the subgroup analyses of the PACIFIC reported improved OS and PFS in patients who started durvalumab within 14 days after CRT as compared to those who started after 14 days. This fact raised the question of whether sequencing and timing could have a role in the response to immunotherapy. This question was partially answered with the results of another study: the LUN 14–179. This single-arm phase II trial examined consolidation therapy with pembrolizumab 4–8 weeks after CRT in 92 patients with stage IIIA and IIIB NSCLC [77]. In terms of results, a median PFS of 17 months was achieved, with a 2-year PFS of 44.6% and 2-year OS rate of 68.7%. Treatment was well tolerated, with 6.5% of grade 3–5 pneumonitis [78]. The fact that these results were comparable to those in the PACIFIC showed that consolidation immunotherapy could also be effective if administered with a certain delay after CRT.

The results of these studies have led to the development of numerous new trials testing different ICI, such as the ongoing RTOG 3505 phase III trial with concurrent CRT followed by nivolumab [79].

### 6.2. Does Immunotherapy Have a Role as Part of Definitive Therapy?

The favorable results of durvalumab as consolidation therapy in NSCLC have motivated the development of new studies that test the use of ICI concurrently to radical treatment (Table 4). However, one of the main concerns of this approach was the possible increase in toxicity. This safety profile was assessed in the ETOP NICOLAS phase II trial, which added nivolumab to standard CRT. 21 patients with stage IIIA-B NSCLC received definitive CRT with both concomitant and maintenance nivolumab. No grade ≥ 3 pneumonitis was reported in the interim analysis, which led to the recruitment of a total of 80 patients. Of these, only 8 experienced grade ≥ 3 pneumonitis [80,81]. One-year OS in the starting cohort was 79%, while the larger cohort is still under evaluation [82].

These promising results were reinforced by those of atezolizumab in this same setting in the phase II trial DETERRED. The experimental arm showed better results in terms of 1-year PFS (57% vs. 50), although 1-year OS was the same in the two groups (79%). When comparing both regimes, no significant increase in toxicity was reported [83].

**Table 4:** Prospective studies that combine RT and ICI in stage III NSCLC.

Trial	Phase	N	Stage	RT Dose (Gy)	ICI Agent	IT Sequence	ORR (%)	OS (%)	PFS	Toxicity ≥ G3 (%)
PACIFIC [8] (Randomized)	3	714	Unresectable III	54–66	Durvalumab	Consolidation	28.4%	1-yr: 83.1 2-yr: 66.3	Median 18.8 months	30.5
LUN 14–179 [77]	2	92	Unresectable IIIA/B	59.4–66	Pembrolizumab	Consolidation	NR	1-yr: 80.5 2-yr: 68.7	Median 15.4 months	6.5
ETOP NICOLAS [82]	2	80	Unresectable IIIA/B	66	Nivolumab	Concurrent + Consolidation	NR	1-yr: 79	1-yr: 54%	10.9
DETERRED [83]	2	40	Unresectable III	60–66	Atezolizumab	Concurrent + Consolidation	NR	1-yr: 79	1-yr: 57%	27.5

RT: radiotherapy. ICI: immune checkpoint inhibitors. ORR: overall response rate. OS: overall survival. PFS: progression-free survival. NR: not reported.

### 6.3. Can immunotherapy Take the Place of Chemotherapy in Definitive Therapy?

Interestingly, several ongoing studies are testing if CT can be replaced by ICI, based on the superior results of immunotherapy in trials, such as the KEYNOTE-024 [7,84]. The NRG Oncology LU004 ARCHON-1 trial will treat 24 patients with PD-L1 > 50% with definitive RT and concurrent durvalumab. Moreover, the DART trial will enroll patients who are unfit for concurrent CRT and administer standard RT with concurrent and consolidation durvalumab.

### 6.4. Is the Neoadjuvant Setting a Good Fit for Immunoradiotherapy?

While no definitive evidence is available yet regarding the clinical efficacy of neoadjuvant immunoradiotherapy in the neoadjuvant setting, various ongoing studies should shed some light in the coming years (Table 5). For now, the only preliminary data available at the moment comes from ICI and CT. For instance, the NADIM is an ongoing phase II trial that combines neoadjuvant CT and nivolumab in resectable stage IIIA N2 patients prior to surgery, followed by adjuvant nivolumab for 1 year. Preliminary results show that, out of the 46 patients included, 41 underwent surgery and all a R0 resection was achieved in all cases. Thirty-five patients achieved a major pathologic response, with 25 of them being complete pathologic responses. Treatment was well tolerated, and 1-year PFS was a promising 95.7% [85].

Although no clinical results are available at the moment, further studies testing the combination of neoadjuvant immunotherapy, CT, and RT are ongoing, such as the phase I CASE 4516 and the phase II NCT03237377 trials.

## 7. Early Stage NSCLC and Small-Cell Lung Cancer: Future Directions for Immunoradiotherapy

Although most of the evidence that supports the combination of RT and ICI in lung cancer comes from the experience in metastatic and stage III NSCLC, multiple trials are currently assessing the efficacy of this approach in other settings.

In the case of early stage NSCLC, there is a relatively high risk of distant recurrence even with the best surgery or SABR (60–80% in node-negative tumors) [86]. For this reason, several trials are addressing if adjuvant ICI after SABR could improve PFS. Along with various phase I and II studies, there is a phase III trial, the PACIFIC 4 that will randomize 630 patients to receive SABR with or without two years of adjuvant durvalumab. Moreover, other trials will test neoadjuvant ICI followed by SABR, such as the PEMBRO-X trial with neoadjuvant pembrolizumab.

The recent approval of ICI as second-line therapy in SCLC will also provide opportunities to test the possible benefits of its combination with RT. The available data suggest that the addition of RT in this setting is probably a safe approach and could improve disease control [87]. For instance, a phase I study that tested the toxicity profile of pembrolizumab and thoracic RT after induction CT in 35 patients with extensive-stage SCLC only reported 2% grade 3 side effects [88]. Further ongoing trials may shed some light in this matter in the years to come.

## 8. Optimizing the Efficacy of Immunoradiotherapy in Lung Cancer

### 8.1. RT Fractionation and ICI Agent

SABR, with high doses per fx, seems to be more immunogenic than conventional RT, given that a daily delivery of RT might kill migrating lymphocytes [89]. However, it has been reported that a radiation dose superior to 10–12 Gy can remove the trigger from the STING pathway, which leads to immunosuppression. The STING pathway participates in the secretion of IFN-I, which has been associated with the AE. High doses of radiation can upregulate the nuclease TREX1, which inhibits this pathway, therefore hindering the immune response [90]. On the other hand, other studies have shown no depletion in immune effector cells after a single dose of 12 Gy, so this might not be the only mechanism involved [91].

The ideal fractionation is also under debate. Generally speaking, protocols of RT delivered in 5 × 6 Gy and 3 × 8 Gy have shown better responses than a single fx [92]. In the recent PEMBRO-RT study, AEs were achieved after 3 × 8 Gy [59]. However, other studies have reported successful results by delivering single doses [93]. Moreover, most clinical trials deliver RT every other day rather than consecutively, based on the idea that it takes 48 h to replenish lymphocytes [32].

There is still no evidence of a possible difference in RT efficacy between anti-PD1/L1 and anti-CTLA-4 agents. A retrospective analysis of the study by Chen et al. [94] showed similar results in metastatic NSCLC patients treated with anti-CTLA-4 or anti-PD-1, if slightly better in the anti-PD-1 cohort.

### 8.2. RT and ICI Sequence

Even though many ongoing trials are delivering concomitant therapy, sequential treatment has also been reported as effective, for instance, in the study by Bauml et al., where they administered pembrolizumab 4–12 weeks after local treatment [60]. Some publications have also suggested that sequencing may depend on the ICI agent [95]. Anti-PD-L1 therapy seems to be more effective when administered concurrently with RT [96], whereas anti-CTLA-4 appears to have better synergy if administered before RT [97]. These differences might be explained by the fact that anti-PD-1/L1 act on newly activated and exhausted T cells [42], whereas anti-CTLA-4 act on naïve T cells and Treg [43]. Some of these uncertainties might be answered in ongoing clinical trials, such as the SABRseq study, in which patients will be assigned to a regimen of either SABR followed by pembrolizumab or pembrolizumab followed by SABR. Moreover, a trial by Davis et al. will divide patients in three treatment arms: concurrent, induction, or sequential atezolizumab and SABR [98].

### 8.3. Number of Irradiated Lesions and Tumor Location

While most clinical trials irradiate a single lesion [9], some publications suggest that multisite irradiation should be the norm, as this would result in a wider variety of TAAs being presented to effector T cells [39]. As a matter of fact, a single-arm phase II study in which 51 patients with NSCLC were treated with local ablative therapies (RT or surgery) to all metastatic sites plus sequential pembrolizumab reported an impressive PFS of 19.1 months and a 1-year OS of 90%, which further supports this multisite approach [60]. Moreover, partial irradiation can also induce AE in cases of bulky tumors where RT to the whole volume would not be feasible [99].

Tumor location also seems to be important. Preclinical studies have suggested that bone lesions are less prone to unleash an AE compared to visceral tumors [100]. Moreover, it is unclear if RT of the lymph nodes can negatively affect the development of an AE. For instance, a preclinical study by Marciscano et al. showed that elective nodal irradiation decreased the efficacy of I-SABR [101]. In contrast, Tang et al. found higher levels of CD8+ T cells after irradiation of the liver compared to other sites [53]. Recent studies have also investigated the influence of the gut microbiota in the immune response to cancer [102]. Whether irradiation to the bowels can play a role in this pathway is still unclear.

### 8.4. Biomarkers

Predictors of response to I-SABR remains a widely unexplored field. Still, some recent studies are showing promising results. For instance, high levels of TGF-β have been associated with worse outcomes, and its blockade with therapies, like fresolimumab, has shown better antitumoral responses [103,104,105]. Furthermore, levels of IFN-γ have been linked to RT effectiveness [106], as well as IFN-I, due its involvement in the STING pathway [38]. These mechanisms, however, are far for simple, as other investigators have shown that persistent high levels of IFN can actually induce resistance to radiation and anti-CTLA-4 treatment [107]. The utility of liquid biopsies is also being investigated, as some trials are measuring the levels of circulating tumor DNA (ctDNA) and other circulating molecules after RT [108,109]. Finally, a recent analysis of three phase I/II trials has found that lymphopenia might negatively impact the chances of obtaining an AE [110]. Key points to consider in a treatment with I-SABR are summarized in Table 6. In short, further studies are needed in order to find biomarkers that can improve patient selection and outcomes.

## 9. Conclusions

ICI have been a breakthrough in the treatment of lung cancer. However, only a limited number of patients benefit from these agents in monotherapy due to resistance mechanisms. For this reason, the combination of RT and ICI is gaining acceptance as a way to overcome these resistances. The evidence discussed in this review suggests that RT is able to restore the efficacy of ICI in non-responding metastatic patients by unleashing an immune systemic response or AE. In stage III NSCLC, the use of immunoradiotherapy has confirmed a significant impact in survival, while its role in early stages is already being evaluated in multiple clinical trials. All this data should make clinicians reconsider the role of RT in patients receiving ICI for all stages of lung cancer and establish immunoradiotherapy as a standard in clinical practice. Its use reduces tumor size and alleviates symptoms but also increases the release of TAAs, delays the time to a new line of systemic treatment, and can improve disease control through the AE. Future studies should prioritize the analysis of different treatment variables to answer the current questions on timing, sequencing, radiation dose, fractionation, and biomarkers in order to optimize treatment efficacy and patient selection.

## Figures and Tables

**Table 1:** Current FDA-approved indications for lung cancer treatment.

Indication	Agent	Use	Line
Unresectable, stage III NSCLC	Durvalumab	Monotherapy	Adjuvant after radical chemo-radiotherapy
Metastatic NSCLC	Pembrolizumab	Combination with platinum + pemetrexed	1st line
		Monotherapy	2nd line
	Nivolumab	Monotherapy	2nd line
	Atezolizumab	Combination with carboplatin + paclitaxel + bevacizumab	1st line
		Monotherapy	2nd line
Metastatic squamous NSCLC	Pembrolizumab	Combination with carboplatin + paclitaxel/nab-paclitaxel	1st line
		Monotherapy	1st line, PD-L1 ≥ 50%
Metastatic SCLC	Atezolizumab	Combination with carboplatin + etoposide	1st line
	Pembrolizumab	Monotherapy	3rd line
	Nivolumab	Monotherapy	3rd line

NSCLC: Non-Small-Cell Lung Cancer. SCLC: Small-Cell Lung Cancer. PD-L1: Programmed death-ligand 1.

**Table 3:** Ongoing clinical trials in patients with metastatic NSCLC receiving ICI and RT.

Trial	Phase	ICI Agent	Design	RT Dose	Primary Endpoint(s)
NCT03223155	I randomized	Ipilimumab + nivolumab	SABR + ICI Sequential arm Concurrent arm	3–5 fx	Number of serious adverse events
NCT03158883	I	Avelumab	ICI + SABR	50 Gy/5 fx	ORR
NCT02239900	I/II randomized	Ipilimumab	ICI + SABR Multiple arms	50 Gy/4 fx or 60 Gy/10 fx 1–4 lesions	MTD
NCT03176173 RRADICAL	II	Nivolumab, pembrolizumab, atezolizumab	ICI ± SABR	1–10 fx	PFS at 24 weeks
NCT03965468 CHESS	II	Durvalumab	ICI + CT + RT	1–10 fx	PFS at 12 months
NCT03867175	III randomized	Pembrolizumab	ICI ± SABR (all metastatic lesions)	3–10 fx	PFS

ICI: immune checkpoint inhibitors. SABR: stereotactic ablative radiotherapy. RT: radiotherapy. Fx: fraction. PFS: progression-free survival. ORR: overall response rate. CT: chemotherapy. MTD: maximum tolerated dose.

**Table 5:** Ongoing clinical trials evaluating the combination of ICI and RT in stage III NSCLC patients.

Study	Phase	N	Stage	RT Dose	ICI Agent	ICI Sequence	Status
CASE4516 NCT02987998	1	20	Resectable IIIA	45 Gy/25 fx	Pembrolizumab	Neoadjuvant + Adjuvant	Active, not recruiting
CLOVER NCT03509012	1	300	Unresectable III NSCLC, SCLC, H & N	Conventional RT	Durvalumab	Concurrent	Recruiting
NCT03053856	2	37	Resectable IIIA N2	44 Gy/22 fx	Pembrolizumab	Adjuvant	Not yet recruiting
NCT03237377	2	32	Resectable IIIA	45 Gy/25 fx	Durvalumab +/-tremelimumab	Neoadjuvant	Recruiting
LUN 16-081 NCT03285321	2	108	Unresectable IIIA/B	59.4–66.6 Gy	Nivolumab +/- ipilimumab	Consolidation	Recruiting
CHIO3 NCT04062708	2	55	Resectable IIIA/B	54 Gy	Durvalumab	Neoadjuvant + Adjuvant	Not yet recruiting
NCT03871153	2	25	Resectable III N2	45-61.2 Gy/25–34 fx	Durvalumab	Neoadjuvant + Adjuvant	Recruiting
KEYNOTE-799 NCT03631784	2	216	Unresectable III	60 Gy/30 fx	Pembrolizumab	Concurrent + Consolidation	Recruiting
NCT03663166	1/2	50	Unresectable III	60Gy/30 fx	Ipilimumab vs. nivolumab	Concurrent vs. Consolidation	Recruiting
NCT03102242	2	63	Unresectable IIIA/B	60 Gy/30 fx	Atezolizumab	Neoadjuvant	Active, not recruiting
NCT03589547	2	25	III	60 Gy RT followed by 20Gy/2–3 fx SABR	Durvalumab	Consolidation	Recruiting
NCT02572843	2	68	Resectable IIIA N2	Conventional RT if R1–2	Durvalumab	Neoadjuvant + Adjuvant	Active, not recruiting
PACIFIC 2 NCT03519971	3	300	Unresectable III	Conventional RT	Durvalumab	Concurrent +/- Consolidation	Active, not recruiting
PACIFIC 5 NCT03706690	3	360	Unresectable III	Conventional RT	Durvalumab	Consolidation	Recruiting
PACIFIC 6 NCT03693300	2	150	Unresectable III	Conventional RT	Durvalumab	Consolidation	Recruiting
MK-3475 NCT03379441	2	126	Unresectable IIIA/IIIB	Conventional RT	Pembrolizumab	Consolidation	Not recruiting

ICI: immune checkpoint inhibitor. RT: radiotherapy. Fx: fraction. NSCLC: non-small cell lung cancer. SCLC: Small cell lung cancer. H&N: head and neck.

**Table 6:** Key questions for a treatment with immunoradiotherapy.

Which RT technique is more immunogenic?	SABR rather than conventional RT. Every other day rather than consecutive. 6–12 Gy per fraction rather than higher doses. 24 Gy/3 fx and 30 Gy/5 fx are the most frequent in clinical trials.
What is the ideal treatment sequence?	Concurrent RT with Anti-PD-1/L1. Sequential RT after Anti-CTLA-4.
Which lesions should be treated?	Multisite irradiation rather than single site. Visceral lesions rather than bone. RT to the lymph nodes and bowel could be detrimental. Partial irradiation of bulky tumors can also unleash AEs.
Are there any biomarkers that can guide patient selection?	High TGF- β has been associated with worse outcomes. High IFN-I/γ could influence RT effectiveness Lymphopenia could negatively impact the immunogenicity of RT. Currently, no biomarkers are approved for use in clinical practice.

RT: radiotherapy. SABR: stereotactic ablative radiotherapy. Fx: fraction. AEs: abscopal effects. TGF- β: transforming growth factor beta. IFN-I/γ: interferon type I/gamma.

## References

[1] Siegel R.L., Miller K.D., Jemal A. (2017). Cancer Stadistics 2017. Ca Cancer J. Clin..

[2] Choi J.L., Simone C.B. (2019). Stereotactic body radiation therapy versus surgery for early stage non-small cell lung cancer: Clearing a path through an evolving treatment landscape. J. Thorac. Dis..

[3] Bradley J.D., Paulus R., Komaki R., Masters G., Blumenschein G., Schild S., Bogart J., Hu J., Forster K., Magliocco A. (2015). Standard-dose versus high-dose conformal radiotherapy with concurrent and consolidation carboplatin plus paclitaxel with or without cetuximab for patients with stage IIIA or IIIB non-small-cell lung cancer (RTOG 0617): A randomized, two-by-two factorial phase 3 study. Lancet Oncol..

[4] Juan O., Popat S. (2017). Ablative Therapy for Oligometastatic Non-Small Cell Lung Cancer. Clin. Lung Cancer.

[5] Schonewolf C.A., Verma V., Post C.M., Berman A.T., Frick M.A., Vachani A., Lin C., Simone C.B. (2018). Outcomes of invasive mediastinal nodal staging versus positron emission tomography staging alone for early-stage non-small cell lung cancer treated with stereotactic body radiation therapy. Lung Cancer.

[6] Evans T., Ciunci C., Hertan L., Gomez D. (2017). Special topics in immunotherapy and radiation therapy: Reirradiation and palliation. Transl. Lung Cancer Res..

[7] Reck M., Rodríguez-Abreu D., Robinson A.G., Hui R., Csöszi T., Fülöp A., Gottfried M., Peled N., Tafreshi A., Cuffe S. (2016). Pembrolizumab versus Chemotherapy for PD-L1- Positive Non-Small-Cell Lung Cancer. N. Engl. J. Med..

[8] Antonia S.J., Villegas A., Daniel D., Vicente D., Murakami S., Hui R., Yokoi T., Chiappori A., Lee K.H., de Wit M. (2017). Durvalumab after chemoradiotherapy in stage III non-small-cell lung cancer. N. Engl. J. Med..

[9] Chicas-Sett R., Morales-Orue I., Castilla-Martinez J., Zafra-Martin J., Kannemann A., Blanco J., Lloret M., Lara P.C. (2019). Stereotactic ablative radiotherapy combined with immune checkpoint inhibitors reboots the immune response assisted by immunotherapy in metastatic lung cancer: A systematic review. Int. J. Mol. Sci..

[10] National Comprehensive Cancer Network^®^ (2020). NCCN Clinical Practice Guidelines in Oncology (NCCN Guidelines^®^): Non-Small Cell Lung Cancer Version 6. https://www.nccn.org/professionals/physician_gls/pdf/nscl.pdf.

[11] Ferlay J., Steliarova-Foucher E., Lorlet-Tieulent J., Rosso S., Coebergh J.W., Comber H., Forman D., Bray F. (2013). Cancer incidence and mortality patterns in Europe: Estimates for 40 countries in 2012. Eur. J. Cancer.

[12] Dandekar V.K., Young J., Kiel K., Bonomi P., Fidler M.J., Batus M., Sher D. (2015). Efficacy and Tolerability of Palliative Split-Course Thoracic Chemoradiotherapy for Symptomatic Non-Small Cell Lung Cancer. Am. J. Clin. Oncol..

[13] Hellman S., Weichselbaum R.R. (1995). Oligometastases. J. Clin. Oncol..

[14] Couñago F., Luna J., Guerrero L.L., Vaquero B., Guillen-Sacoto M.C., Gonzalez-Merino T., Taboada B., Diaz V., Rubio-Viqueira B., Diaz-Gavela T. (2019). Management of oligomtastatic non-small cell lung cancer patients: Current controversies and future directions. World J. Clin. Oncol..

[15] Gomez D.R., Tang C., Zhang J., Blumenschein G.R., Hernandez M., Lee J.J., Ye R., Palma D.A., Louie A.V., Camidge D.R. (2019). Local consolidative therapy vs. maintenance therapy or observation for patients with oligometastatic non-small-cell lung cancer: Long-term results of a multi-institutional, phase II, randomized study. J. Clin. Oncol..

[16] Palma D.A., Olson R., Harrow S., Gaede S., Louie A.V., Haasbeek C., Mulroy L., Lock M., Rodriguez G.B., Yaremko B.P. (2020). Stereotactic ablative radiotherapy for the comprehensive treatment of oligometastatic cancers: Long-term results of the SABR-COMET phase II randomized trial. J. Clin. Oncol..

[17] Aupérin A., Péchoux C.L., Rolland E., Curran W.J., Furuse K., Fournel P., Belderbos J., Clamon G., Ulutin H.C., Paulus R. (2010). Meta-analysis of concomitant versus sequential radiochemotherapy in locally advanced non-small-cell lung cancer. J. Clin. Oncol..

[18] Spigel D.R., Reckamp K.L., Rizvi N.A., Poddubskaya E., West H.J., Eberhardt W.E.E., Baas P., Antonia S.J., Pluzanski A., Vokes E.E. (2015). A phase III study (CheckMate 017) of nivolumab (NIVO; anti-programmed death-1 [PD-1]) vs. docetaxel (DOC) in previously treated advanced or metastatic squamous (SQ) cell non-small cell lung cancer (NSCLC). J. Clin. Oncol..

[19] Paz-Ares L., Horn L., Borghaei H., Spigel D.R., Steins M., Ready N., Quan L., Chow M., Vokes E.E., Felip E. (2015). Phase III, randomized trial (CheckMate 057) of nivolumab (NIVO) versus docetaxel (DOC) in advanced non-squamous cell (non-SQ) non-small cell lung cancer (NSCLC). J. Clin. Oncol..

[20] Herbst R.S., Baas P., Kim D.W., Felip E., Pérez-Gracia J.L., Han J.Y., Molina J., Kim J.H., Arvis C.D., Ahn M.J. (2016). Pembrolizumab versus docetaxel for previously treated, PD-L1-positive, advanced non-small-cell lung cancer (KEYNOTE-010): A randomised controlled trial. Lancet.

[21] Rittmeyer A., Barlesi F., Waterkamp D., Park K., Ciardiello F., von Pawel J., Gadgeel S.M., Hida T., Kowalski D.M., Cobo Dols M. (2017). Atezolizumab versus docetaxel in patients with previously treated non-small-cell lung cancer (OAK): A phase 3, open-label, multicentre randomised controlled trial. Lancet.

[22] Paz-Ares L., Luft A., Vicente D., Tafreshi A., Gümüş M., Mazières J., Hermes B., Senler F.C., Csöszi T., Fülöp A. (2018). Pembrolizumab plus chemotherapy for squamous non-small-cell lung cancer. N. Engl. J. Med..

[23] Borghaei H., Langer C.J., Gadgeel S.M., Papadimitrakopoulou V.A., Patnaik A., Powell S.F., Gentzler R.D., Martins R.G., Stevenson J.P., Jalal S.I. Pemetrexed-Carboplatin Plus Pembrolizumab as First-Line Therapy for Advanced Nonsquamous NSCLC: KEYNOTE-021 Cohort G Update. Proceedings of the IASLC 18th World Conference on Lung Cancer.

[24] Socinski M.A., Jotte R.M., Cappuzzo F., Orlandi F., Stroyakovskiy D., Nogami N., Rodriguez-Abreu D., Moro-Sibilot D., Thomas C.A., Barlesi F. (2018). Atezolizumab for First-Line Treatment of Metastatic Nonsquamous NSCLC. N. Engl. J. Med..

[25] Horn L., Mansfield A.S., Szczȩsna A., Havel L., Krzakowski M., Hochmair M.J., Huemer F., Losonczy G., Johnson M.L., Nishio M. (2018). First-line atezolizumab plus chemotherapy in extensive-stage small-cell lung cancer. N. Engl. J. Med..

[26] Haanen J.B.A.G., van Thienen H., Blank C.U. (2015). Toxicity patterns with immunomodulating antibodies and their combinations. Semin. Oncol..

[27] Voskens C.J., Goldinger S.M., Loquai C., Robert C., Kaehler K.C., Berking C., Bergmann T., Bockmeyer C.L., Eigentler T., Fluck M. (2013). The price of tumor control: An analysis of rare side effects of anti-CTLA-4 therapy in metastatic melanoma from the ipilimumab network. PLoS ONE.

[28] Connell P.P., Hellman S. (2009). Advances in radiotherapy and implications for the next century: A historical perspective. Cancer Res..

[29] Baskar R., Lee K.A., Yeo R., Yeoh K.-W. (2012). Cancer and radiation therapy: Current advances and future directions. Int. J. Med. Sci..

[30] Yazan A., Puja V., Sungjune K. (2016). Systematic review of case reports on the abscopal effect. Curr. Probl. Cancer.

[31] Mole R.H. (1953). Whole Body Irradiation—Radiobiology or Medicine?. Br. J. Radiol..

[32] Demaria S., Golden E.B., Formenti S.C. (2015). Role of Local Radiation Therapy in Cancer Immunotherapy. JAMA Oncol..

[33] Grass G.D., Krishna N., Kim S. (2016). The immune mechanisms of abscopal effect in radiation therapy. Curr. Probl. Cancer.

[34] Hwang W.L., Pike L.R.G., Royce T.J., Mahal B.A., Loeffler J.S. (2018). Safety of combining radiotherapy with immune-checkpoint inhibition. Nat. Rev. Clin. Oncol..

[35] Sharabi A.B., Nirschl C.J., Kochel C.M., Nirschl T.R., Francica B.J., Velarde E., Deweese T.L., Drake G.D. (2015). Stereotactic radiation therapy augments antigen-specific PD-1-mediated antitumor immune responses via cross-presentation of tumor antigen. Cancer Immunol. Res..

[36] Gupta A., Probst H.C., Vuong V., Landshammer A., Muth S., Yagita H., Schwendener R., Pruschy M., Knuth A., van den Broek M. (2012). Radiotherapy Promotes Tumor-Specific Effector CD8 + T Cells via Dendritic Cell Activation. J. Immunol..

[37] Barker H.E., Paget J.T.E., Khan A.A., Harrington K.J. (2015). The tumour microenvironment after radiotherapy: Mechanisms of resistance and recurrence. Nat. Rev. Cancer.

[38] Liang H., Deng L., Hou Y., Meng X., Huang X., Rao E., Zheng W., Maureci H., Mack M., Xu M. (2017). Host STING-dependent MDSC mobilization drives extrinsic radiation resistance. Nat. Commun..

[39] Brooks E.D., Chang J.Y. (2019). Time to abandon single-site irradiation for inducing abscopal effects. Nat. Rev. Clin. Oncol..

[40] Demaria S., Kawashima N., Yang A.M., Devitt M.L., Babb J.S., Allison J.P., Formenti S.C. (2005). Immune-mediated inhibition of metastases after treatment with local radiation and CTLA-4 blockade in a mouse model of breast cancer. Clin. Cancer Res..

[41] Vatner R.E., Cooper B.T., Vanpouille-Box C., Demaria S., Formenti S.C. (2014). Combinations of immunotherapy and radiation in cancer therapy. Front. Oncol..

[42] Spranger S., Gajewski T.F. (2018). Mechanisms of Tumor Cell–Intrinsic Immune Evasion. Annu. Rev. Cancer Biol..

[43] Buchbinder E.I., Desai A. (2016). CTLA-4 and PD-1 pathways similarities, differences, and implications of their inhibition. Am. J. Clin. Oncol. Cancer Clin. Trials.

[44] Wao H., Mhaskar R., Kumar A., Miladinovic B., Djulbegovic B. (2013). Survival of patients with non-small cell lung cancer without treatment: A systematic review and meta-analysis. Syst. Rev..

[45] Borghaei H., Paz-Ares L., Horn L., Spigel D.R., Steins M., Ready N.E., Chow L.Q., Vokes E.E., Felip E., Holgado E. (2015). Nivolumab versus docetaxel in advanced nonsquamous non-small-cell lung cancer. N. Engl. J. Med..

[46] Fehrenbacher L., Spira A., Ballinger M., Kowanetz M., Vansteenkiste J., Mazieres H., Park K., Smith D., Artal-Cortes A., Lewanski C. (2016). Atezolizumab versus docetaxel for patients with previously treated non-small-cell lung cancer (POPLAR): A multicenter, open-label, phase 2 randomised controlled trial. Lancet.

[47] Chicas-Sett R., Morales-Orue I., Rodriguez-Abreu D., Lara-Jimenez P. (2018). Combining radiotherapy and ipilimumab induces clinically relevant radiation-induced abscopal effects in metastatic melanoma patients: A systematic review. Clin. Transl. Radiat. Oncol..

[48] Torok J.A., Salama J.K. (2019). Combining immunotherapy and radiotherapy for the STR treatment. Nat. Rev. Clin. Oncol..

[49] Sharvedian N., Lisberg A.E., Bornazyan K., Veruttipong D., Goldman J.W., Formenti S.C., Garon E.B., Lee P. (2017). Previous radiotherapy and the clinical activity and toxicity of pembrolizumab in the treatment of non-small-cell lung cancer: A secondary analysis of the KEYNOTE-001 phase 1 trial. Lancet Oncol..

[50] Desideri I., Francolini G., Scotti V., Pezzulla D., Becherini C., Terziani F., Paoli C.D., Olmetto E., Visani L., Meattini I. (2019). Benefit of ablative versus palliative-only radiotherapy in combination with nivolumab in patients affected by metastatic kidney and lung cancer. Clin. Transl. Oncol..

[51] Ratnayake G., Shanker M., Roberts K., Mason R., Hughes B.G.M., Lwin Z., Jain V., O’Byrne K., Lehman M., Chua B. (2020). Prior or concurrent radiotherapy and nivolumab immunotherapy in non-small lung cancer. Asia Pac. J. Clin. Oncol..

[52] Samstein R., Rimner A., Barker C.A., Yamada Y. (2017). Combined immune checkpoint blockade and radiation therapy: Timing and dose fractionation associated with greatest survival duration among over 750 treated patients. Int. J. Radiat. Oncol. Biol. Phys..

[53] Tang C., Welsh J.W., de Groot P., Massarelli E., Chang J.Y., Hess K.R., Basu S., Curran M.A., Cabanillas M.E., Subbiah V. (2017). Ipilimumab with stereotactic ablative radiation therapy: Phase I results and immunologic correlates from peripheral T cells. Clin. Cancer Res..

[54] Formenti S.C., Rudqvist N.-P., Golden E., Cooper B., Wennerberg E., Lhuillier C., Vanpouille-Box C., Friedman K., De Andrade L.F., Wucherpfennig K.W. (2018). Radiotherapy induces responses of lung cancer to CTLA-4 blockade. Nat. Med..

[55] Welsh J.W., Tang C., de Groot P., Naing A., Raju U., Shaaban S., Chang J.Y., Cushman T., Heymach J., Dadu R. (2017). Phase II 5-arm trial of ipilimumab plus lung or liver stereotactic radiation for patients with advanced malignancies. Int. J. Radiat. Oncol. Biol. Phys..

[56] Chicas-Sett R., Morales-Orue I., Castilla-Martinez J.F., Blanco J., Kannemann A., Zafra J., Zajac M., Lloret M., Lara P.C. (2019). I-SABR induces local and abscopal responses in metastatic patients after failure to ICI treatment. Radiother. Oncol..

[57] Theelen W., Peulen H., Lalezari F., van der Noort V., de Vries J.F., Aerts J., Dumoulin D.W., Bahce I., Niemeijer A.N., de Langen A.J. (2019). Effect of Pembrolizumab After Stereotactic Body Radiotherapy vs. Pembrolizumab Alone on Tumor Response in Patients With Advanced Non-Small Cell Lung Cancer: Results of the PEMBRO-RT Phase 2 Randomized Clinical Trial. JAMA Oncol..

[58] Welsh J.W., Menon H., Tang C., Verma V., Altan M., Hess K.R., de Groot P., Nguyen Q., Simon G.R., Ferdinandos Skoulidis F. (2019). Randomized phase I/II trial of pembrolizumab with and without radiotherapy for metastatic non-small cell lung cancer. J. Clin. Oncol..

[59] Patel J.D., Bestvina C.M., Karrison T., Jelinek M.J., Juloori A., Pointer K., Hoffman P.C., Pitroda S.P., Vokes E.E., Chmura S.J. (2020). Randomized phase I trial to evaluate Concurrent or Sequential ipilimumab, nivolumab, and stereotactic body radiotherapy in patients with stage IV non-small cell lung cancer (COSINR study). J. Clin. Oncol..

[60] Bauml J.M., Mick R., Ciunci C., Aggarwal C., Davis C., Evans T., Deshpande C., Miller L., Patel P., Alley E. (2019). Pembrolizumab After Completion of Locally Ablative Therapy for Oligometastatic Non–Small Cell Lung Cancer. JAMA Oncol..

[61] Cheema P.K., Rothenstein J., Melosky B., Brade A., Hirsh V. (2019). Perspectives on treatment advances for stage III locally advanced unresectable non-small-cell lung cancer. Curr. Oncol..

[62] Kumar S.S., Higgins K.A., McGarry R.C. (2017). Emerging Therapies for Stage III Non-Small Cell Lung Cancer: Stereotactic Body Radiation Therapy and Immunotherapy. Front. Oncol..

[63] Albain K.S., Swann R.S., Rusch V.W., Turrisi A.T., Shepherd F.A., Smith C., Chen Y., Livingston R.B., Feins R.H., Gandara D.R. (2009). Radiotherapy plus chemotherapy with or without surgical resection for stage III non-small cell lung cancer: A phase III randomised controlled trial. Lancet.

[64] Curran W.J., Paulus R., Langer C.J., Komaki R., Lee J.S., Hauser S., Movsas B., Wasserman T., Rosenthal S.A., Gore E. (2011). Sequential vs. concurrent chemoradiation for stage III non-small cell lung cancer: Randomized phase III trial RTOG 9410. J. Natl. Cancer Inst..

[65] Vokes E.E., Herndon J.E., Kelley M.J., Cicchetti M.G., Ramnath N., Neill H., Atkins J.N., Watson D.M., Akerley W., Green M.R. (2007). Induction chemotherapy followed by chemoradiotherapy compared with chemoradiotherapy alone for regionally advanced unresectable stage III non-small-cell lung cancer: Cancer and leukemia group B. J. Clin. Oncol..

[66] Hanna N., Neubauer M., Yiannoutsos C., McGarry R., Arseneau J., Ansari R., Reynolds C., Govindan R., Melnyk A., Fisher W. (2008). Phase III study of cisplatin, etoposide, and concurrent chest radiation with or without consolidation docetaxel in patients with inoperable stage III non-small-cell lung cancer: The Hoosier Oncology Group and U.S.. Oncol. J. Clin. Oncol..

[67] Huber R.M., Flentje M., Schmidt M., Pöllinger B., Gosse H., Willner J., Ulm K., Bronchial Carcinoma Therapy Group (2006). Simultaneous chemoradiotherapy compared with radiotherapy alone after induction chemotherapy in inoperable stage IIIA or IIIB non-small-cell lung cancer: Study CTRT99/97 by the Bronchial Carcinoma Therapy Group. J. Clin. Oncol..

[68] Flentje M., Huber R.M., Engel-Riedel W., Andreas S., Kollmeier J., Staar S., Dickgreber N., Vaissiere N., De Almeida C., Edlich B. (2016). GILT--A randomised phase III study of oral vinorelbine and cisplatin with concomitant radiotherapy followed by either consolidation therapy with oral vinorelbine and cisplatin or best supportive care alone in stage III non–small cell lung cancer. Strahlenther. Onkol..

[69] Ahn J.S., Ahn Y.C., Kim J.H., Lee C.G., Cho E.K., Lee K.C., Chen M., Kim D.W., Kim H.K., Min Y.J. (2015). Multinational randomized phase III trial with or without consolidation chemotherapy using docetaxel and cisplatin after concurrent chemoradiation in inoperable stage III non–small-cell lung cancer: KCSG-LU05-04. J. Clin. Oncol..

[70] Kelly K., Chansky K., Gaspar L.E., Albain K.S., Jett J., Ung Y.C., Lau D.H., Crowley J.J., Gandara D.R. (2008). Phase III trial of maintenance gefitinib or placebo after concurrent chemoradiotherapy and docetaxel consolidation in inoperable stage III non-smallcell lung cancer: SWOG S0023. J. Clin. Oncol..

[71] Butts C., Socinski M.A., Mitchell P.L., Thatcher N., Havel L., Krzakowski M., Nawrocki S., Ciuleanu T.E., Bosquée L., Trigo J.M. (2014). Tecemotide (L-BLP25) versus placebo after chemoradiotherapy for stage III non-small-cell lung cancer (START): A randomised, double-blind, phase 3 trial. Lancet Oncol..

[72] Giaccone G., Bazhenova L.A., Nemunaitis J., Tan M., Juhász E., Ramlau R., van den Heuvel M.M., Lal R., Kloecker G.H., Eaton K.D. (2015). A phase III study of belagenpumatucel-L, an allogeneic tumour cell vaccine, as maintenance therapy for non–small cell lung cancer. Eur. J. Cancer.

[73] Senan S., Brade A., Wang L.H., Vansteenkiste J., Dakhil S., Biesma B., Martinez Aguillo M., Aerts J., Govindan R., Rubio-Viqueira B. (2016). PROCLAIM: Randomized phase III trial of pemetrexed–cisplatin or etoposide–cisplatin plus thoracic radiation therapy followed by consolidation chemotherapy in locally advanced nonsquamous non-small-cell lung cancer. J. Clin. Oncol..

[74] Antonia S.J., Villegas A., Daniel D., Vicente D., Murakami S., Hui R., Kurata T., Chiappori A., Lee K.H., de Wit M. (2018). Overall survival with durvalumab after chemoradiotherapy in stage III NSCLC. N. Engl. J. Med..

[75] Botticella A., Mezquita L., Le Pechoux C., Planchard D. (2019). Durvalumab for stage III non-small-cell lung cancer patients: Clinical evidence and real-world experience. Ther. Adv. Respir. Dis..

[76] Gray J.E., Villegas A., Daniel D., Vicente D., Murakami S., Hui R., Kurata T., Chiappori A., Lee K.H., Cho B.C. (2019). Three-year overall survival update from the PACIFIC trial. J. Clin. Oncol..

[77] Durm G.A., Althouse S.K., Sadiq A.A., Jalal S.I., Jabbour S., Zon R., Kloecker G.H., Fisher W.B., Reckamp K.L., Kio E.A. (2018). Phase II trial of concurrent chemoradiation with consolidation pembrolizumab in patients with unresectable stage III non–small cell lung cancer: Hoosier Cancer Research Network LUN 14-179. J. Clin. Oncol..

[78] Durm G.A., Althouse S.K., Sadiq A.A., Jalal S.I., Jabbour S., Zon R., Kloecker G.H., Fisher W.B., Reckamp K.L., Kio E.A. (2018). Updated Results of a Phase II Trial of Concurrent Chemoradiation with Consolidation Pembrolizumab in Patients with Unresectable Stage III NSCLC. J. Thorac. Oncol..

[79] Gerber D.E., Urbanic J.J., Langer C., Hu C., Chang I.F., Lu B., Movsas B., Jeraj R., Curran W.J., Bradley J.D. (2017). Treatment design and rationale for a randomized trial of cisplatin and etoposide plus thoracic radiotherapy followed by nivolumab or placebo for locally advanced non–small-cell lung cancer (RTOG 3505). Clin. Lung Cancer.

[80] Peters S., Felip E., Dafni U., Belka C., Guckenberger M., Irigoyen A., Nadal E., Becker A., Vees H., Pless M. (2019). Safety evaluation of nivolumab added concurrently to radiotherapy in a standard first line chemo-radiotherapy regimen in stage III non-small cell lung cancer-The ETOP NICOLAS trial. Lung Cancer.

[81] Fitzgerald K., Simone C.B. (2020). Combining Immunotherapy with Radiation Therapy in Non-Small Cell Lung Cancer. Thorac. Surg. Clin..

[82] Peters S., Felip E., Dafni U., Tufman A., Guckenberger M., Irigoyen A., Nadal E., Becker A., Vees H., Pless M. (2019). Efficacy evaluation of concurrent nivolumab addition to a first-line, concurrent chemo-radiotherapy regimen in unresectable locally advanced NSCLC: Results from the European Thoracic Oncology Platform (ETOP 6–14) NICOLAS phase II trial. Ann. Oncol..

[83] Lin S.H., Lin Y., Mok I., Young J.A., Phan S., Sandler A., Papadimitrakopoulou V., Heymach J., Tsao A.S. (2018). DETERRED: Phase II trial combining atezolizumab concurrently with chemoradiation therapy in locally advanced non-small cell lung cancer. J. Thorac. Oncol..

[84] Reck M., Rodríguez-Abreu D., Robinson A.G., Hui R., Csőszi T., Fülöp A., Gottfried M., Peled N., Tafreshi A., Cuffe S. (2019). Updated Analysis of KEYNOTE-024: Pembrolizumab Versus Platinum-Based Chemotherapy for Advanced Non-Small-Cell Lung Cancer With PD-L1 Tumor Proportion Score of 50% or Greater. J. Clin. Oncol..

[85] Provencio M., Nadal E., Insa A., Campelo R.G., Casal J., Domine M., Majem M., Rodriguez-Abreu D., Martinez-Marti A., De Castro J. (2019). OA13.05 NADIM Study: Updated Clinical Research and Outcomes. J. Thorac. Oncol..

[86] Senthi S., Lagerwaard F.J., Haasbeek C.J., Slotman B.J., Senan S. (2012). Patterns of disease recurrence after stereotactic ablative radiotherapy for early stage non-small-cell lung cancer: A retrospective analysis. Lancet Oncol..

[87] Nesbit E.G., Leal T.A., Kruser T.J. (2019). What is the role of radiotherapy for extensive-stage small cell lung cancer in the immunotherapy era?. Transl. Lung Cancer Res..

[88] Welsh J.W., Heymach J.V., Chen D., Verma V., Cushman T.R., Hess K.R., Shroff G., Tang C., Skoulidis F., Jeter M. (2020). Phase I Trial of Pembrolizumab and Radiation Therapy after Induction Chemotherapy for Extensive-Stage Small Cell Lung Cancer. J. Thorac. Oncol..

[89] Garelli E., Rittmeyer A., Putora P.M., Glatzer M., Dressel R., Andreas S. (2019). Abscopal effect in lung cancer: Three case reports and a concise review. Immunotherapy.

[90] Vanpouille-Box C., Alard A., Aryankalayil M.J., Sarfraz Y., Diamond J.M., Schneider R.J., Inghirami G., Coleman C.N., Formenti S.C., Demaria S. (2017). DNA exonuclease Trex1 regulates radiotherapy-induced tumour immunogenicity. Nat. Commun..

[91] Siva S., MacManus M.P., Martin R.F., Martin O.A. (2015). Abscopal effects of radiation therapy: A clinical review for the radiobiologist. Cancer Lett..

[92] Dewan M.Z., Galloway A.E., Kawashima N., Dewyngaert J.K., Babb J.S., Formenti S.C., Demaria S. (2009). Fractionated but not single-dose radiotherapy induces an immune-mediated abscopal effect when combined with anti-CTLA-4 antibody. Clin. Cancer Res..

[93] Ngwa W., Irabor O.C., Schoenfeld J.D., Hesser J., Demaria S., Formenti S.C. (2018). Using immunotherapy to boost the abscopal effect. Nat. Rev. Cancer.

[94] Chen D., Menon H., Verma V., Guo C., Ramapriyan R., Barsoumian H., Younes A., Hu Y., Wasley M., Cortez M.A. (2020). Response and outcomes after anti-CTLA4 versus anti-PD1 combined with stereotactic body radiation therapy for metastatic non-small cell lung cancer: Retrospective analysis of two single-institution prospective trials. J. Immunother. Cancer.

[95] Buchwald Z.S., Wynne J., Nasti T.H., Zhu S., Mourad W.F., Yan W., Gupta S., Khleif S.N., Khan M.K. (2018). Radiation, immune checkpoint blockade and the abscopal effect: A critical review on timing, dose and fractionation. Front. Oncol..

[96] Dovedi S.J., Adlard A.L., Lipowska-Bhalla G., McKenna C., Jones S., Cheadle E.J., Stratford I.J., Poon E., Morrow M., Stewart R. (2014). Acquired resistance to fractionated radiotherapy can be overcome by concurrent PD-L1 blockade. Cancer Res..

[97] Young K.H., Baird J.R., Savage T., Cottam B., Friedman D., Bambina S., Messenheimer D.J., Fox B., Newell P., Bahjat K.S. (2016). Optimizing timing of immunotherapy improves control of tumors by hypofractionated radiation therapy. PLoS ONE.

[98] MPDL3280A and Stereotactic Ablative Radiotherapy in Patients with Non-Small Cell Lung Cancer. https://ClinicalTrials.gov/show/NCT02400814.

[99] Tubin S., Popper H.H., Brcic L. (2019). Novel stereotactic body radiation therapy (SBRT)-based partial tumor irradiation targeting hypoxic segment of bulky tumors (SBRT-PATHY): Improvement of the radiotherapy outcome by exploiting the bystander and abscopal effects. Radiat. Oncol..

[100] Farach A., Farach-Carson M.C., Butler E.B., Chang J.C., Teh B.S. (2015). The role of combined radiation and immunotherapy in breast cancer treatment. J. Radiat. Oncol..

[101] Marciscano A.E., Ghasemzadeh A., Nirschl T.R., Theodros D., Kochel C.M., Francica B.J., Muroyama Y., Anders R.A., Sharabi A.B., Velarde E. (2018). Elective nodal irradiation attenuates the combinatorial efficacy of stereotactic radiation therapy and immunotherapy. Clin. Cancer Res..

[102] Routy B., Le Chatelier E., Derosa L., Duong C., Alou M.T., Daillère R., Fluckiger A., Messaoudene M., Rauber C., Roberti M.P. (2018). Gut microbiome influences efficacy of PD-1–based immunotherapy against epithelial tumors. Science.

[103] Vanpouille-Box C., Diamond J.M., Pilones K.A., Zavadil J., Babb J.S., Formenti S.C., Barcellos-Hoff M.H., Demaria S. (2015). TGFβ Is a Master Regulator of Radiation Therapy-Induced Antitumor Immunity. Cancer Res..

[104] Formenti S.C., Lee P., Adams S., Goldberg J.D., Li X., Xie M.W., Ratikan J.A., Felix C., Hwang L., Faull K.F. (2018). Focal Irradiation and Systemic TGFβ Blockade in Metastatic Breast Cancer. Clin. Cancer Res..

[105] Rodríguez-Ruiz M.E., Rodríguez I., Mayorga L., Labiano T., Barbes B., Etxeberria I., Ponz-Sarvise M., Azpilikueta A., Bolaños E., Sanmamed M.F. (2019). TGFβ Blockade Enhances Radiotherapy Abscopal Efficacy Effects in Combination with Anti-PD1 and Anti-CD137 Immunostimulatory Monoclonal Antibodies. Mol. Cancer Ther..

[106] Gerber S.A., Sedlacek A.L., Cron K.R., Murphy S.P., Frelinger J.G., Lord E.M. (2013). IFN-γ mediates the antitumor effects of radiation therapy in a murine colon tumor. Am. J. Pathol..

[107] Benci J.L., Xu B., Qiu Y., Wu T.J., Dada H. (2016). Tumor Interferon Signaling Regulates a Multigenic Resistance Program to Immune Checkpoint Blockade. Cell.

[108] Abbosh C., Birkbak N.J., Wilson G.A., Jamal-Hanjani M., Constantin T., Salari R., Le Quesne J., Moore D.A., Veeriah S., Rosenthal R. (2017). Phylogenetic ctDNA analysis depicts early-stage lung cancer evolution. Nature.

[109] Adams D.L., Adams D.K., He J., Kalhor N., Zhang M., Xu T., Gao H., Reuben J.M., Qiao Y., Komaki R. (2017). Sequential tracking of PD-L1 expression and RAD50 induction in circulating tumor and stromal cells of lung cancer patients undergoing radiotherapy. Clin. Cancer Res..

[110] Chen D., Verma V., Patel R.R., Barsoumian H.B., Cortez M.A., Welsh J.W. (2020). Absolute Lymphocyte Count Predicts Abscopal Responses and Outcomes in Patients Receiving Combined Immunotherapy and Radiotherapy: A prospective-retrospective analysis of 3 phase I/II Trials. Int. J. Radiat. Oncol. Biol. Phys..

